# Content of n-3 LC-PUFA in Breast Milk Four Months Postpartum is Associated with Infancy Blood Pressure in Boys and Infancy Blood Lipid Profile in Girls

**DOI:** 10.3390/nu11020235

**Published:** 2019-01-22

**Authors:** Signe Bruun, Lenie van Rossem, Lotte Lauritzen, Steffen Husby, Lotte Neergaard Jacobsen, Kim F. Michaelsen, Maria Boysen Sandberg, Ken D. Stark, Jan Sørensen, Gitte Zachariassen

**Affiliations:** 1 Strategic Business Unit Pediatric, Arla Foods Ingredients Group P/S, DK-8260 Viby J, Denmark; lotte.neergaard.jacobsen@arlafoods.com; 2 Hans Christian Andersen Children’s Hospital, Odense University Hospital, DK-5000 Odense C, Denmark; steffen.husby@rsyd.dk (S.H.); gitte.zachariassen@rsyd.dk (G.Z.); 3 Department of Clinical Research, Faculty of Health Sciences, University of Southern Denmark, DK-5000 Odense C, Denmark; 4 Odense Patient data Explorative Network (OPEN), Odense University Hospital, DK-5000 Odense C, Denmark; 5 Julius Center for Health Sciences and Primary Care, Public Health Epidemiology, University Medical Center Utrecht, NL-3584 CG Utrecht, The Netherlands; l.vanrossem@umcutrecht.nl; 6 Department of Nutrition, Exercise and Sports, University of Copenhagen, DK-1958 Frederiksberg C, Denmark; ll@nexs.ku.dk (L.L.); kfm@nexs.ku.dk (K.F.M.); 7 Department of Clinical Pharmacology, Odense University Hospital, DK-5000 Odense C, Denmark; maria.sandberg@rsyd.dk; 8 Department of Kinesiology, Faculty of Applied Health Sciences, University of Waterloo, Waterloo, ON N2L 3G1, Canada; kstark@uwaterloo.ca; 9 Danish Centre for Health Economics (DaCHE), Department of Public Health, University of Southern Denmark, DK-5000 Odense C, Denmark; jansorensen@rcsi.ie; 10 Healthcare Outcome Research Centre, Royal College of Surgeons in Ireland, Dublin D02 YN77, Ireland

**Keywords:** cohort study, cardiovascular health, omega-3, fatty acids, human milk, milk composition, cholesterol, triglyceride

## Abstract

Blood pressure (BP) and blood lipid profile (BLP) have been shown to track from childhood into adulthood, and n-3 long-chain polyunsaturated fatty acids (LC-PUFAs) in breast milk have been suggested as mediators of the beneficial long-term effect of breastfeeding on BP and BLP. We aimed to investigate associations between n-3 LC-PUFA content in breast milk at 4 months postpartum and offspring BP and BLP in early life. BP and BLP were measured at 4, 18, and 36 months. Statistical analyses were sex-stratified and adjusted for gestational age, maternal pre-pregnancy body mass index (BMI), and maternal educational level. Based on 336 mother-child dyads, high n-3 LC-PUFA in breast milk was inversely associated with systolic and diastolic BP in boys at 4 months (β = −20.0 (95% CI = −33.4, −6.7), *p* = 0.004 and β = −10.2 (95% CI = −19.8, −0.5), *p* = 0.039, respectively); inversely associated with HDL cholesterol, and directly associated with triglyceride in girls at 4 months (β = −0.7 (95% CI = −1.1, −0.3), *p* = 0.001 and β = 3.1 (95% CI = 1.0, 5.2), *p* = 0.005, respectively). Associations observed at the later time points were non-significant. Furthermore, we observed sex-specific changes over time in both size and direction of the associations. Our results indicate that early intake of n-3 LC-PUFA can affect early development in cardiometabolic factors such as BP and BLP in a sex-specific manner. Follow-up and further investigation in later childhood is planned.

## 1. Introduction

The evidence of blood pressure (BP) tracking from childhood to adulthood is strong, and BP trajectories associated with risk of cardiovascular disease (CVD) are detectable already in childhood [1]. Elevated childhood BP is considered a predictor of (early) adult hypertension and likewise, normal BP in childhood is associated with lack of hypertension in adulthood [2]. Similarly, blood lipid profile (BLP; i.e., total cholesterol, high density lipoprotein (HDL) cholesterol, low density lipoprotein (LDL) cholesterol, and triglycerides) in childhood is strongly correlated with adulthood BLP, especially total cholesterol [3], for which the correlation is even stronger than the one for BP [4].

Both breastfeeding duration and exclusivity have been suggested as potential modifiers of the risk of later CVD, and breastfeeding is part of the World Health Organization’s (WHO) “Global Action Plan for the prevention and control of noncommunicable diseases 2013–2020” [5]. Results on associations between breastfeeding and later CVD risk are conflicting, but a WHO systematic review and meta-analysis by Horta and Victora from 2013 found a small, protective (lowering) effect of breastfeeding on systolic BP (SBP) in adulthood [6], but in an updated version of the review from 2015 (independent of the WHO), 48 recent studies were included. The authors found no association between breastfeeding and SBP or diastolic BP (DBP), and no association between breastfeeding and total cholesterol, and it was suggested that earlier estimates were affected by publication bias [7], a concern raised by others as well [8].

Several mechanisms have been suggested to explain a possible protective effect of breastfeeding on later elevated BP, e.g., protection against childhood obesity which is related to the development of future hypertension [9,10,11]. However, the association between breastfeeding and obesity is weak and not likely an important mediator of the effect of breastfeeding on BP [6]. Another mechanism suggested is the (postnatal) growth acceleration hypothesis, which proposes that infant formula feeding increases the fat mass in late infancy [12,13], programming an abnormal vascular biology associated with early CVD [14].

Associations between breastfeeding and BLP in infancy, in particular total cholesterol and LDL cholesterol, are considered a direct consequence of the nutrients intake from breast milk and infant formula, respectively, i.e., differences in the lipid profile and fatty acid composition of the milk. A plausible biological mechanism behind a potential nutritional programming effect of breastmilk on BLP in later life is unclear [8,15]; but down-regulation of the hepatic hydroxymethylglutaryl coenzyme A (HMG-CoA) and thereby reduced cholesterol synthesis is suggested [6].

Differences in lipid composition of breast milk and infant formula are also suggested as an explanatory factor, especially the difference in the content of essential n-3 and n-6 polyunsaturated fatty acids (PUFAs). Standard infant formula contains only α-linolenic acid (ALA; C18:3 (n-3)) and linoleic acid (LA; C18:2 (n-6)). Breast milk, on the other hand, contains long-chain (LC)-PUFAs, mainly docosahexaenoic acid (DHA; C22:6 (n-3)), eicosapentaenoic acid (EPA; C20:5 (n-3)), and arachidonic acid (AA; C20:4 (n-6)) [6]. These LC-PUFAs are also endogenously synthesized from ALA and LA; a synthesis upregulated in pregnant women and young infants [16,17]. Even though the adding of LC-PUFAs to infant formula has evolved in the last decades, the effects on growth, cognitive development, and CVD risk markers continue to be debated [6,18,19].

In infancy, the main source of dietary DHA and EPA is breast milk, whereas the main source later in life is fatty fish such as mackerel, herring, and salmon [17]. The concentration of DHA in breast milk is highly varying depending on maternal fish intake [20], and the optimal level is unknown, but based on clinical trials it has been suggested, that approx. 0.3% (weight percentage (wt%)) may be a relevant target [21].

In adults, n-3 and n-6 LC-PUFAs have been shown to affect both BP and BLP [22], whereas few studies have examined the effect in infancy. Some studies indicate that associations between n-3 LC-PUFAs and BP and BLP (as well as neurodevelopmental outcomes) in infants and young children may be sex-specific [23,24,25]. In the present study, we investigated sex-specific associations between the total n-3 LC-PUFAs content (defined as DHA and EPA) in breast milk and offspring BP and BLP in infancy and preschool childhood.

## 2. Materials and Methods

### 2.1. Participants

For consistency, the terms “child” and “children” are used regardless of age, and any time designation such as weeks or months refers to time postpartum, i.e., the child’s age.

This study was embedded in the Odense Child Cohort, an unselected birth cohort comprising children born in the municipality of Odense, Denmark. The cohort is described in detail elsewhere [26], but to summarize, pregnant women were invited to participate from January 2010 to December 2012, i.e., children were born between August 2010 and October 2013. From March 2012, the inclusion was extended to 2.5 months postpartum, but the majority of participants were included during pregnancy. The only exclusion criterion was emigration from the municipality of Odense before birth. 

Mothers completed self-administered questionnaires, and the children were seen for physical examinations at 4, 18, and 36 months. The cohort is ongoing and further self-administered questionnaires and physical examinations are planned at 5, 7, 9, 12, 15, and 18 years.

Background information on maternal age at parturition, maternal pre-pregnancy BMI, postdelivery parity, maternal educational level, maternal smoking during pregnancy, birth type, child’s sex, gestational age, and birth weight were available from cohort questionnaires or medical hospital records. Information on current breastfeeding status at time of milk sample delivery and duration of breastfeeding was available from cohort questionnaires or weekly text message questions [27].

### 2.2. Breast Milk Fatty Acids

From May 2012, mothers were asked to provide a breast milk sample, when their child was seen for the first physical examination approx. 4 months postpartum. A total amount of 30 mL was required, but less was accepted. No requirements or recordings regarding the sample being fore- or hind milk or the use of a breast pump were specified. The sample was split into 10 mL tubes (100 × 16PP, Sarstedt, Nümbrecht, Germany) and stored at 5 °C. If the full amount of 30 mL was provided, one of the 10 mL tubes was stored at −20 °C for later macronutrients analysis. Within three days after sample collection, the tubes stored at 5 °C were centrifuged at 3600 rpm and 21 °C for five minutes (Eppendorf Centrifuge 5702 R, Eppendorf Corporate, Wesseling-Berzdorf, Germany). The resulting fat, skimmed, and solid fractions were manually aliquoted (3.5 mL transfer pipette, Sarstedt, Nümbrect, Germany) into three different tubes (3.6 mL Nunc^®^ CryoTubes^®^, Thermo Fisher Scientific, Waltham, MA, USA) and stored at −80 °C. The fat fractions were shipped on dry ice from Odense, Denmark to Waterloo, Canada, and remained frozen upon arrival.

The fatty acid composition of the fat fraction was determined by high throughput gas chromatography [28]. Briefly, lipids were extracted from 50 µL of the sample using 2:1 (*v*/*v*) chloroform:methanol containing an internal standard (19:0 methyl ester, Nu-Chek Prep, Inc., Elysian, MN, USA) and butyl-hydroxytoluene as an antioxidant. The samples were vortexed briefly, and the organic and aqueous phases were separated by the addition of 0.2 M sodium phosphate buffer. The bottom organic layer was collected, and the total lipid extract was dried under nitrogen gas. Samples were reconstituted in hexane, and fatty acid methyl esters were generated by transesterification using 14% boron trifluoride in methanol and convectional heating at 100 °C for one hour. The hexane layer (containing the fatty acid methyl esters) was collected and analysed using a Varian^®^ 3900 gas chromatograph equipped with a DB-FFAP 15 m × 0.10 mm injected dose × 0.10 μm film thickness, nitroterephthalic acid modified, polyethylene glycol, capillary column (J&W Scientific from Agilent Technologies, Mississauga, ON, Canada), set to a 300:1 split ratio. The carrier gas was hydrogen, and a temperature ramping protocol to maximize peak resolution was used [28]. Fatty acid methyl esters were identified by retention time matched to an external standard (GLC-462, Nu-Chek Prep, Inc., Elysian, MN, USA). Output data was expressed quantitatively (μg fatty acid/μL breast milk) through the use of the internal standard, and qualitatively as the fatty acid weight percentage of total fatty acids (wt%).

### 2.3. Blood Pressure Measurement

At 4, 18, and 36 months, an automated, oscillometric device (Welch Allyn Connex^®^ Vital Signs Monitor 6000 Series™, Skaneateles Falls, NY, USA) was used. From 1 February 2016, both oscillometric and auscultatory technique were used at 36 months, the latter by the use of a mercury sphygmomanometer (Riester empire^®^ N, Rudolf Riester GmbH, Jungingen, Germany) in combination with an ultrasound Doppler (UltraTec PD1v, Ultrasound Technologies Ltd., Caldicot, UK). In the present study, only the oscillometric measurements are included.

At 4 and 18 months (the latter until April 15th 2013), measurements were performed in a supine position, thereafter in a sitting position. At all ages, measurements were performed on the left upper arm; after measurement of head and abdominal circumferences, but prior to venipuncture. One measurement was performed per child and device. The cuff sizes available were; 9–13 and 12–16 cm (4 months), 12–16 and 15–21 cm (18 months), 13–17 and 15–21 cm (36 months, manual and automated, respectively).

All measurements were performed by trained personnel with years of experience working with children. The setting of the examinations and BP measurements was office-like and “cozy” with the personnel dressed in civilian clothes.

### 2.4. Blood Pressure Z-Scores and Percentiles

Converting an absolute BP value (mmHg) to a Z-score or percentile takes into account sex, age, length or height of the child, and provides a precise classification of BP according to body size, thus avoiding the misclassification of children who are very tall or short [9,29].

Regarding children <1 year, the normative BP values (in terms of Z-scores) published in the 1987 guideline are still recommended as the reference [30]. The resulting Z-scores were converted to percentiles in the present study.

For children >1 year, an update of the 2004 guideline was published in September 2017 [9]. In the updated version, the normative data (in terms of percentiles) are based on auscultatory BP measurements, and children and adolescents being overweight or obese are excluded. Noteworthy, the 2017 update uses height instead of height-for-age Z-score (HAZ), based on the assumption that BP is a function of height and not its’ Z-score [31]. The equations used in the 2017 update are available online as a Statistical Analysis System (SAS) macro [32]. For the present study, a STATA do-file was created, available from the authors on request. 

In children >1 year, normal BP is categorized as the 50th percentile, elevated BP as >90th percentile, stage 1 hypertension as ≥95th percentile and stage 2 hypertension as ≥95th percentile + 12 mmHg [9].

### 2.5. Blood Lipid Profile

In relation to the physical examinations, non-fasting venous blood samples were drawn if the parents accepted. At the 36 months’ examination, all children were offered local dermal analgesia (AMETOP Gel 4%, Smith & Nephew, Chippenham, UK) [33]. Blood samples were collected between 8 am and 5 pm. Samples were collected in BD Vacutainer^®^ PST™ Gel Separator Tube with lithium heparin (Thermo Fisher Scientific, Waltham, MA, USA); centrifuged, separated, and aliquoted within four hours of collection. The blood samples were analyzed as normal routine tests on Abbott Architect instruments, c8000 or c16000 (Abbott, Green Oaks, IL, USA). The instruments were calibrated and used according to the manufacturer’s instructions. LDL cholesterol values were obtained using the Friedewald equation [34], unless triglyceride was > 4 mmol/L, in which case LDL cholesterol was direct-measured. All samples tested at the Architect system underwent automatized analysis for hemolysis, icterus, and lipemia/turbidity interference (HIL-index). 

Routine screening is not recommended, and adverse BLP concentrations consequently not defined for children <2 years. In children between 2 and 8 years, screening is only recommended in case of family history of dyslipidemia or premature CVD [35]. In this case, total cholesterol ≥ 5.2 mmol/L (200 mg/dL), LDL cholesterol ≥ 3.4 mmol/L (130 mg/dL), or triglycerides ≥ 1.1 mmol/L (100 mg/dL) are defined as high concentrations. In the case of HDL cholesterol, concentrations < 1.0 mmol/L (40 mg/dL) are defined as low [35].

### 2.6. Ethics

The study was approved by the Danish Data Protection Agency (ref. 12/26892) and The Regional Committees on Health Research Ethics for Southern Denmark (ref. S-20090130, sub-protocols 12, 18, and 37). The study complied with the Declaration of Helsinki II including written, informed consent from participants.

### 2.7. Statistics

Descriptive statistics were performed, and basic characteristics of the following groups are presented:

- Those attending the physical examination at 4 months and delivering a breast milk sample (*n* = 336)
- Those attending the physical examination at 4 months, but not delivering a breast milk sample (*n* = 1967)
- Those not attending the physical examination at 4 months (*n* = 359)

Data are presented as mean ± standard deviation (SD) if normally distributed, otherwise as median (interquartile range (IQR)). Graphical inspections were used to assess assumptions of normal distribution. Categorical variables were compared using χ^2^ test, and continuous variables were compared using Welsh’s two-sample t-test (if normally distributed), or two-sample Kolmogorov-Smirnov equality-of-distributions test (if not normally distributed). 

Breast milk fatty acid content is presented as relative values, i.e., the fatty acid as weight percentage of total fatty acid content (wt%), treated as a continuous variable. Kernel density estimates, P-P plots, and Q-Q plots were used to assess assumptions of normal distribution of residuals.

We made overall analyses with test for interaction between sex and n-3 LC-PUFA as well as sex-stratified analyses. As the primary inferential analysis, linear regression was used to determine (cross sectional) associations between total n-3 LC-PUFA in breast milk at 4 months and infant BP or BLP at 4, 18, and 36 months, respectively. Secondary, supplementary analyses were performed for DHA and EPA as individual exposures, i.e., associations between DHA and EPA (in turn) and infant BP or BLP at 4, 18, and 36 months, respectively, were investigated. Furthermore, multilevel analyses were used to examine time and n-3 LC-PUFA interactions. All analyses were adjusted for gestational age in days (continuous), maternal pre-pregnancy BMI in kg/m^2^ (continuous), and educational level (three categories). The selection of variables included as covariates was based on the construction of a directed acyclic graph (graph not shown). 

Level of significance was set at α < 0.05. Statistics were performed using STATA IC/15.1 (TX, USA).

## 3. Results

### 3.1. Participants

**Figure 1:**
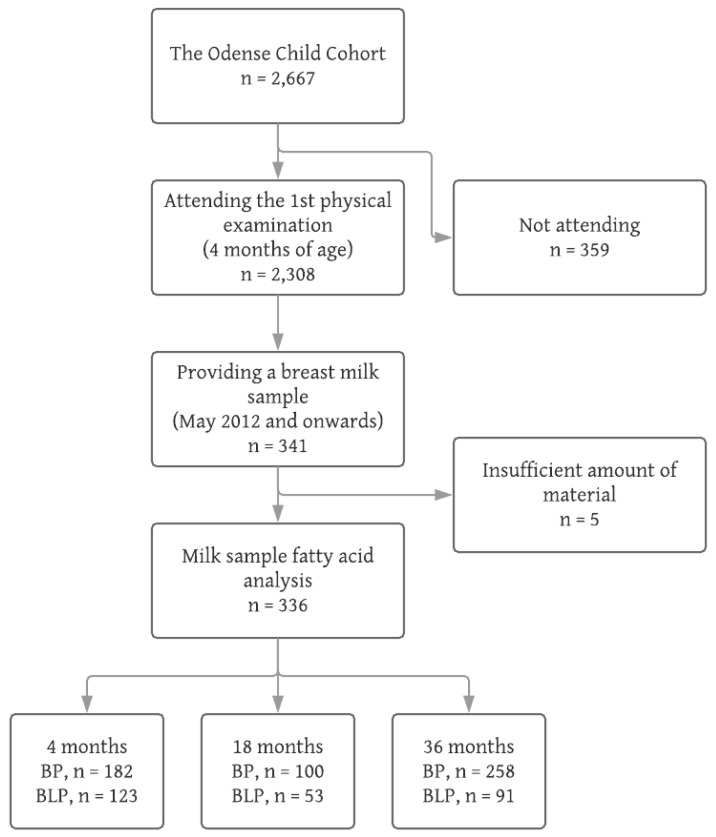
Flowchart of inclusion. Breast milk samples were not collected before May 2012. BP = blood pressure measurement, BLP = blood lipid profile on venous blood sample.

At the first physical examination, a total of 336 mothers provided a milk sample of sufficient amount for the fatty acid analysis. A flowchart of the inclusion is shown in Figure 1. 

Of the 336 children, 27 (8.0%) attended the first examination only, 110 (32.7%) attended the first and one more (at either 18 or 36 months), and 199 (59.2%) attended all three examinations. At time of milk sample delivery, a total of 136 (40.5%) of the 336 children were exclusively breastfed; 132 (39.3%) were partially breastfed; and data on breastfeeding exclusivity were missing for the remaining 68 (20.2%).

Children’s and maternal characteristics are shown in Table 1. Compared to the mother-child dyads who did not deliver a breast milk sample (group B) and the mother-child dyads who did not attend the first examination (group C), more participating mothers (group A) had higher educational level (*p* < 0.001); fewer were smoking (*p* < 0.001); and fewer had delivered by Caesarean section (*p* = 0.002). Additionally, participating mothers (group A) were older than mothers not attending (group C: *p* = 0.003); and their children had larger birth weight (group B: *p* = 0.001, group C: *p* < 0.001), higher BWZ (group B: *p* = 0.023, group C: *p* = 0.030), and higher gestational age (group C: *p* = 0.004)).

### 3.2. Breast Milk Fatty Acids

A total of 96.78 ± 0.67 wt% (mean ± SD) of the fatty acids in the breast milk samples was identified. Saturated fatty acids (SFAs) and monounsaturated fatty acids (MUFAs) comprised more than two thirds of the total fatty acids in the breast milk samples, whereas PUFAs comprised approx. 14% (wt%), and n-3 LC-PUFAs 0.3% (wt%). DHA accounted for approx. 75% of the total n-3 LC-PUFAs. Overall, no differences in fatty acid composition were observed between boys and girls (Table 2).

Breast milk samples from 7 mothers (three boys’ and four girls’ mothers) had an n-3 LC-PUFA > 1.0% (wt%), and this high n-3 LC-PUFA content was found to correlate with a low overall content of fat in the breast milk samples, since 5 of the 7 had a total breast milk fat content <50th percentile, and the remaining 2 had no data on macronutrients composition.

### 3.3. Blood Pressure and Blood Lipid Profile

Boys had a higher SBP at 4 months (103.8 mmHg versus 99.6 mmHg, *p* = 0.028), and a higher DBP percentile at 36 months (92th versus 86th, *p* < 0.001) compared to girls (Table 3). No other differences were observed in SBP or DBP at any of the other time points.

Noteworthy, 29.3% of the boys and 20.0% of the girls had a SBP > 90th percentile, and 39.1% of the boys and 41.6% of the girls had a DBP > 90th percentile at 4 months. At 18 months, this increased to 50.0% of the boys and 63.6% of the girls for SBP and for DBP; 87.5% and 79.5% of the boys and girls, respectively. However, the prevalence decreased again at 36 months, where 20.8% of the boys and 20.3% of the girls had a high SBP and 67.7% of the boys and 43.0% of the girls had high DBP.

No differences were observed in BLP between boys and girls except for triglycerides at 18 months of age, where boys had a higher level than girls (1.3 mmol/L versus 1.0 mmol/L, *p* = 0.023).

None of the children had elevated total cholesterol at 36 months, but 2.3% of the girls had elevated LDL; 8.5% of the boys and 25.0% of the girls had low HDL concentration; and 12.8% of the boys and 27.3% of the girls had elevated triglycerides.

### 3.4. Content of n-3 LC-PUFA in Breast Milk and Associations with Infancy Blood Pressure

Overall, we observed no associations between n-3 LC-PUFA in breast milk and BP at any age, but we observed a clear tendency towards an interaction between sex and n-3 LC-PUFA, especially for SBP at 4 and 18 months, where *p* = 0.089 and 0.081 for mmHg and *p* = 0.191 and 0.021 for percentile at 4 and 18 months, respectively.

In boys, high n-3 LC-PUFA in breast milk was inversely associated with SBP at 4 months (*p* = 0.004 and *p* = 0.005, for mmHg and percentile, respectively), whereas non-significant associations in the opposite direction were indicated in girls at 4 months (Table 4). At later time points (18 and 36 months), the directions of the associations were reversed in both boys and girls. At 18 months, n-3 LC-PUFA was inversely associated with SBP in girls, only significant for SBP percentiles (*p* = 0.041), but no significant associations were observed in either boys or girls at 36 months of age.

Although there were no significant interactions between sex and n-3 LC-PUFA for DBP (*p* > 0.10 for all measures), sex-differences were observed in the stratified analyses. Like for SBP, high n-3 LC-PUFA was inversely associated with DBP at 4 months in boys (only significant for absolute DBP (*p* = 0.039)), and non-significant associations in the opposite direction were indicated in girls (Table 4). Again, the directions of the associations tended to be reversed at 18 and 36 months in both boys and girls, but none of them reached significance.

Excluding the children of the mothers with a breast milk n-3 LC-PUFA >1.0 wt% did not change the associations for SBP or DBP, although slightly changing the level of significance (SBP at 4 months in boys, (mmHg) β = −21.8 (95% CI = −40.0, −3.6), *p* = 0.020 and (percentile) β = −31.4 (95% CI = −64.7, 1.9), *p* = 0.064; SBP at 18 months in girls, (percentile) β = −41.8 (95% CI = −63.8, −19.7), *p* = 0.001).

The associations between n-3 LC-PUFA and SBP were furthermore confirmed by the multilevel analyses; with 4 months as the reference, the estimates for the time and n-3 LC-PUFA interaction were β = 11.2 (95% CI = −6.7, 29.2), *p* = 0.220 at 18 months and β = 13.5 (95% CI = 1.1, 25.9), *p* = 0.033 at 36 months in boys and β = -11.9 (95% CI = −28.8, 4.9), *p* = 0.166 and β = 0.0 (95% CI = −11.9, 11.9), *p* > 0.9, respectively, in girls. The estimates for DBP at 18 and 36 months with 4 months as the reference were β = 4.1 (95% CI = −9.9, 18.1), *p* = 0.566 and β = 9.5 (95% CI = −0.2, 19.2), *p* = 0.054, respectively, in boys and β = −9.5 (95% CI = −24.6, 5.6), *p* = 0.216 and β = 0.2 (95% CI = −10.4, 10.9), *p* = 0.963 in girls.

The patterns of associations observed in both the cross sectional and the multilevel analyses were retained when breast milk EPA and DHA were included individually (Supplementary Tables S1 and S2).

### 3.5. Content of n-3 LC-PUFA in Breast Milk and Associations with Infancy Blood Lipid Profile

Overall, we observed no associations between n-3 LC-PUFA in breast milk and BLP at any age, but we observed a significant interaction between sex and n-3 LC-PUFA in HDL cholesterol and triglycerides at 4 months (*p* = 0.041 and *p* = 0.038, respectively).

No associations between n-3 LC-PUFA in breast milk and BLP were observed in boys at any of the time points. However, in girls, high n-3 LC-PUFA was associated with low HDL cholesterol (*p* = 0.001) and high triglyceride (*p* = 0.005) at 4 months, but not at the later time points (Table 4). No associations were observed between breast milk n-3 LC-PUFA and total or LDL cholesterol in either boys or girls at any time point.

Excluding the children of the mothers with a breast milk n-3 LC-PUFA >1.0 wt% did not change the findings in boys or the inverse association with HDL cholesterol at 4 months in girls (β = −0.8 (95% CI = −1.3, −0.3), *p* = 0.001), but the association with triglyceride was reduced and did not reach significance (β = 1.8 (95% CI = −0.7, 4.3), *p* = 0.153).

In the multilevel analyses, the associations between n-3 LC-PUFA and HDL cholesterol and triglycerides were confirmed. For HDL cholesterol (4 months as reference), the estimates for the time and n-3 LC-PUFA interaction at 18 and 36 months were β = −0.1 (95% CI = −0.8, 0.5), *p* = 0.699 and β = 0.0 (95% CI = −0.5, 0.6), *p* = 0.860 in boys and β = −0.3 (95% CI = −1.4, 0.8), *p* = 0.605 and β = 0.4 (95% CI = −0.1, 0.9), *p* = 0.152 in girls. For triglycerides (4 months as reference), the estimates for 18 and 36 months were β = 0.3 (95% CI = −2.1, 2.7), *p* = 0.806 and β = 0.5 (95% CI = −1.5, 2.5), *p* = 0.647 in boys and β = −1.4 (95% CI = −6.5, 3.7), *p* = 0.593 and β = −2.5 (95% CI = −4.9, −0.0), *p* = 0.046 in girls. In all analyses, the patterns were retained for EPA and DHA individually (Supplementary Tables S1 and S2).

## 4. Discussion

Based on 336 mother-child dyads from an unselected birth cohort, we determined the fatty acid content in breast milk collected 4 months postpartum and investigated associations between the n-3 LC-PUFA content and offspring BP and BLP at 4, 18, and 36 months. We observed consistent, significant associations between high n-3 LC-PUFA content in breast milk and low SBP and DBP in boys at 4 months; and low HDL cholesterol and high triglyceride in girls at 4 months. These findings were supported by multilevel analyses and analyses for sex interactions.

Although comparisons with other studies are challenged by different ways of reporting, the fatty acid composition in breast milk in our study are generally in line with findings from previous studies [36,37,38,39]. In general, the overall mean level of DHA in breast milk were comparable to the suggested optimal level of 0.3% (wt%) [21]. Seven of the mothers in our study had a breast milk n-3 LC-PUFA content > 1.0% (wt%), which could be due to recent consumption of fatty fish. Breast milk n-3 LC-PUFA is highly variable, so it would have been optimal to collect samples for several days and not just a spot sample. Although recent fish intake is the main cause of variability in breast milk n-3 LC-PUFA content, it has also been shown to be affected by total energy intake and the percentage of energy from fat in mother’s diet [40]. The observed variation could be due to variation in total fat content of the milk, but with the use of fatty acid wt%, this should not be a major problem, although we did find that the breast milk samples with the high n-3 LC-PUFA content had a low total fat content.

In our study, high n-3 LC-PUFA in breast milk was not associated with BP in general, but the associations were modified by sex with low SBP and DBP in boys at 4 months of age and a tendency to high SBP and DBP in girls. In contrast to our findings, SBP was reduced by 6 mmHg at 12 months of age in healthy Danish children after three months of daily fish oil supplementation (924 mg n-3 LC-PUFA/day), but the intervention did not affect DBP and the study did not stratify by sex [41]. A later study with a longer intervention (from 9 to 18 months) did not find any overall effects on SBP or DBP at 18 months, but a sex-specific effect on mean arterial pressure (MAP) reflecting a reduction in boys and a tendency in the opposite direction in girls [42]. A sex-specific effect was also observed in another Danish randomized controlled trial, where lactating mothers from birth to 4 months of age received olive oil or fish oil in a dose that resulted in a breast milk DHA content of approx. 1 wt%, resulting in an infant intake of around 260 mg/day. This early intake of DHA did not give rise to any difference in BP between the groups at 2.5 years follow-up [43], but a sex-specific effect was observed at 7 and 13 years’ follow-up, where DBP was higher in the supplemented group in boys only [44,45]. An Australian randomized controlled trial, where infants were assigned to receive either olive oil or 650 mg n-3 LC-PUFA/day, found no differences in SBP or DBP at 5 years’ follow-up [46]. In contrast, high total n-3 LC-PUFA content in breast milk was associated with low SBP and DBP at 12 years’ follow-up, but this study did not stratify by sex [47].

A large number of children in the present study had elevated BP at the different time points, which could be partly due to use of an oscillometric device. The use of manual auscultatory devices is the preferred option, but automated oscillometric devices are also accepted in children [48]. A systematic review and meta-analysis on the subject in the paediatric population (*n* = 26,879) concluded, that oscillometric (automatic) devices yielded modestly higher SBP (pooled effect estimate 2.53 mmHg (95% CI = 0.57, 4.50)), whereas DBP did not differ (pooled effect estimate 1.55 mmHg (95% CI = −0.20, 3.31)) [49]. Additionally, BP was only measured once per visit, and—despite attempts to calm the children—this first (and only) BP measurement is probably higher than any subsequent measurements would have been (known as the accommodation effect [9]). If the children were less calm during the BP measurements at 18 and 36 months, this could explain, why we observed no associations at these time points. Their calmness should not be related to the n-3 LC-PUFA content in breast milk, although there could be a link between maternal fish intake, maternal child care, and/or the child’s behavior [50]. In rodents, n-3 LC-PUFA deficiency has been shown to decrease stress robustness, mainly in male offspring [51,52,53]. Such an effect could give rise to the sex-specific associations observed in the present, but one could also speculate, that the observed sex-specific changes in the associations over time might be related to differences in cognitive development between boys and girls; e.g., girls being more obstinate or anxious at the 18 and 36 months’ visits. This would give rise to imprecise BP measurements which together with an imprecision in the measured breast milk n-3 LC-PUFA could hide a real association.

We did not find any overall association between breast milk n-3 LC-PUFA and the BLP. However, as for BP, we found sex-specific associations for triglyceride and HDL cholesterol. High n-3 LC-PUFA in breast milk was not associated with BLP in boys, but with low HDL cholesterol and high triglycerides in girls. In contrast to this, the previously mentioned study with three months’ of daily fish oil supplementation (924 mg n-3 LC-PUFA/day) of healthy 9 months old Danish children observed higher total cholesterol and LDL cholesterol at 12 months [41]. In the following trial, fish oil supplementation from 9 to 18 months resulted in reduced triglyceride independent of sex, but no effect on cholesterols [42]. No long-term follow-up of the maternal fish oil supplementation during lactation has been performed, but in the present study, the lack of associations at 18 and 36 months could indicate that an association is only present as long as the children are exposed to n-3 LC-PUFA, i.e., at 4 months in this study. A similar acute effect could also explain the lack of association between breast milk n-3 LC-PUFA and BP, although other studies in children have observed long-term associations. It is reasonable to expect that effects of diet on the BLP are acute, since the BLP is affected post-prandial after intake of fat. This could also explain the observed high prevalence of elevated BLP in the children. Approx. 13% and 9% of the boys and 27% and 25% of the girls had elevated triglyceride or low HDL concentration, respectively, at 36 months. The children were not fasting before blood sampling, as it is problematic to request an overnight fasting. Nevertheless, the impact and necessity of fasting when assessing BLP is debated [54,55], and it is not likely to affect the HDL concentration. Studies in older children have shown effects of fish oil supplementation on BLP in terms of increased HDL (and non-HDL) cholesterol [56]. It is furthermore well-acknowledged that fish oil supplementation reduce triglycerides and increase HDL cholesterol in adults [57]. Many studies in adults have also found that fish oil supplementation reduce BP (both systolic and diastolic) [22,58,59], although no effect on BP was observed in a recent Cochrane review [60].

The main strength of the present study is the well described study population. We did thorough adjustment with inclusion of gestational age, maternal pre-pregnancy BMI, and educational level, but there are several potential confounders that we were not able to take into account. We did not have any information on family history of hypertension and/or food intake beyond infancy. One could speculate that mothers with a high LC-PUFA content in their breast milk had high dietary intake of LC-PUFAs, which also—after introduction to solid foods—could be the case for their child. This could have been accounted for if we had been able to adjust for red blood cell n-3 LC-PUFA in the child at each visit. As in all observational studies, residual confounding is a possibility, and it is uncertain, to what extent the observed associations reflect causality. The sample size varies between visits, which is hard to avoid in research among young children.

The young age of the study population limits the interpretations with respect to long-term consequences and risk of CVD. Based on previous studies, BP measurements at any age between 6 and 18 years have been shown to be predictive of later hypertension [4]. BLP measurement at any age between 3 and 18 years has been shown to be predictive of later dyslipidemia in males, whereas the prediction of dyslipidemia from BLP in females were most pronounced from 12 to 18 years [4]. There is a lack of data on the predictive value of BP and BLP measurements in the age of the present study population. Breastfed infants have been shown to have higher total cholesterol and LDL cholesterol in infancy (>1 year) compared to infant formula fed children, shifting to no difference in later childhood (1–16 years), and lower levels in adulthood [15,61]. It is hypothesized that elevated cholesterol levels in infancy programs a more efficient cholesterol metabolism later in life [61]. To our knowledge, no data or theories describe how early low plasma concentrations of triglyceride or HDL cholesterol may program later BLP. The observed sex-specific association with n-3 LC-PUFA could somehow interfere with the expected tracking, and it is very difficult to predict how this may influence cardiovascular risk in the long-term. Follow-up is planned at later ages, and we will be able to see, to what extent the early sex-specific differences in the present study influence BP and BLP in later childhood and adolescence.

## 5. Conclusions

In conclusion, the content of n-3 LC-PUFA in breast milk at 4 months postpartum was associated with low SBP and DBP in boys and low HDL cholesterol and high triglyceride in girls at 4 months. Our findings are in line with observed sex-specific associations between n-3 LC-PUFA and BP and BLP in previous studies in children. Our results indicate that the direction of associations may vary with age in a sex-specific manner, which warrant further investigation and follow-up in later childhood.

## Figures and Tables

**Table 1:** Basic characteristics Mother-child dyads who (**A**) attended the physical examination at 4 months of age and delivered a breast milk sample of sufficient amount; (**B**) attended the physical examination at 4 months of age without delivering a breast milk sample; and (**C**) did not attend the physical examination at 4 months of age. Numbers in bold indicate values statistically significant different from group A (*p*-values in main text).

Group	A	B	C
*n*	336	1967	359
**Maternal Characteristics**			
Age at parturition, years			
mean (SD)	30.6 (4.1)	30.4 (4.5)	**29.2 (5.0)**
Pre-pregnancy BMI, kg/m^2^			
median (IQR)	22.9 (21.1, 26.1)	23.4 (21.3, 26.4)	23.4 (20.8, 26.3)
Postdelivery parity, *n* (%)			
primiparous	172 (51.2)	1106 (56.2)	205 (57.1)
Parity 2	120 (35.7)	658 (33.5)	112 (31.2)
Parity 3	39 (11.6)	158 (8.0)	32 (8,9)
Parity ≥ 4	4 (1.2)	34 (1.7)	10 (2.8)
unknown	1 (0.3)	11 (0.6)	-
Educational level, *n* (%)			
Low	49 (14.6)	**391 (19.9)**	**95 (26.5)**
Intermediate	154 (45.8)	**741 (37.7)**	**106 (29.5)**
High	74 (22.0)	**329 (16.7)**	**28 (7.8)**
unknown	59 (17.6)	**506 (25.7)**	**130 (36.2)**
Smoking status, *n* (%)			
Smoker	1 (0.3)	**70 (3.6)**	**32 (8.9)**
Nonsmoker ^1^	334 (99.4)	**1882 (95.7)**	**323 (90.0)**
unknown	1 (0.3)	**15 (0.8)**	**4 (1.1)**
Birth type, *n* (%)			
Vaginal	276 (82.1)	**1511 (76.8)**	**254 (70.8)**
Caesarean section	60 (17.9)	**456 (23.8)**	**105 (29.2)**
**Children’s Characteristics**			
Sex, *n* (%)			
Male	175 (52.1)	1025 (52.1)	196 (54.6)
Female	161 (47.9)	942 (47.9)	163 (45.4)
Gestational age, days			
median (IQR)	281 (274, 288)	281 (273, 287)	**279 (271, 286)**
Birth weight, g			
mean (SD)	3575 (487)	**3482 (567)**	**3404 (639)**
unknown	-	3	1
Birth weight Z-score, SD			
mean (SD)	−0.01 (1.0)	**−0.14 (1.0)**	**−0.19 (1.1)**
unknown	-	3	1
Birth weight Z-score group, *n* (%)			
BWZ < −2 SD	6 (1.8)	55 (2.8)	16 (4.5)
−2 SD ≤ BWZ ≤ 2 SD	319 (94.9)	1,863 (94.7)	332 (92.5)
BWZ > 2 SD	11 (3.3)	46 (2.3)	10 (2.8)
unknown	-	3 (0.2)	1 (0.3)

^1^ Or stopped smoking during 1st trimester. SD, standard deviation; IQR, interquartile range.

**Table 2:** Breast milk fatty acid composition. Values are wt% FA of total FA, presented as mean (SD) if normally distributed, otherwise as median (IQR).

	FA wt% of Total FA	
	Girls	Boys
*n*	161	175
Total SFAs	42.49 (5.17)	42.37 (4.14)
Total MUFAs	40.49 (3.89)	40.74 (3.35)
Total PUFAs	13.68 (2.67)	13.41 (2.38)
Total n-3 PUFA	11.84 (2.34)	11.66 (2.22)
Total LA ^1^	10.76 (2.26)	10.58 (2.14)
Total AA	0.37 (0.09)	0.35 (0.08)
Total n-3 PUFA	1.81 (0.61)	1.73 (0.48)
Total ALA ^2^	1.09 (0.85, 1.46)	1.10 (0.86, 1.39)
Total EPA	0.08 (0.06, 0.12)	0.08 (0.06, 0.12)
Total DHA	0.25 (0.19, 0.34)	0.24 (0.17, 0.35)
Total n-3 LC-PUFAs (EPA + DHA)	0.34 (0.25, 0.47)	0.33 (0.24, 0.46)
n-6/n-3 PUFA ratio	6.80 (5.59, 8.03)	6.81 (5.62, 8.15)

^1^ n-6 precursor; ^2^ EPA and DHA precursor. FA, fatty acid; SFAs, saturated fatty acids; MUFAs, monounsaturated fatty acids; PUFAs, polyunsaturated fatty acids; LA, linoleic acid; AA, arachidonic acid; ALA, α-linolenic acid; EPA, eicosapentaenoic acid; DHA, docosahexaenoic acid.

**Table 3:** Blood pressure and blood lipid profile at the three different time points, stratified by sex. Values are presented as mean (SD) if normally distributed, otherwise median (IQR).

	Girls			Boys		
	4 Months	18 Months	36 Months	4 Months	18 Months	36 Months
Attending, *n*	161	98	143	175	115	152
Age, months	4.0 (3.4, 4.4)	18.6 (18.4, 19.1)	36.1 (35.9, 36.5)	4.0 (3.4, 4.4)	18.6 (18.2, 19.2)	36.1 (35.9, 36.5)
**Blood pressure, *n***	90 ^1^	46 ^2^	133 ^3^	92	56	133 ^4^
Age, months	4.1 (3.6, 4.5)	18.7 (18.3, 19.2)	36.1 (35.9, 36.5)	4.0 (3.6, 4.4)	18.6 (18.2, 19.2)	36.1 (35.9, 36.5)
Systolic BP, mmHg	99.6 (11.3)	103.5 (9.5)	98.1 (7.3)	103.8 (14.0)	102.3 (9.1)	98.4 (7.3)
Diastolic BP, mmHg	61.1 (11.1)	63.4 (6.9)	62.3 (5.8)	61.6 (9.6)	63.6 (8.6)	61.8 (5.6)
Systolic BP, percentile	68.2 (23.6)	87.9 (12.7)	72.6 (18.5)	72.2 (25.1)	86.5 (16.3)	74.9 (17.4)
Diastolic BP, percentile	78.3 (23.2)	93.4 (7.4)	86.4 (10.8)	83.3 (16.2)	95.8 (4.9)	91.7 (6.9)
**Blood lipid profile, *n***	63 ^5^	21 ^6^	44	60	32	47
Age, months	4.2 (3.8, 4.4)	18.8 (18.5, 19.2)	36.0 (35.7, 36.3)	4.0 (3.7, 4.5)	18.8 (18.3, 19.6)	36.1 (35.9, 36.6)
Total cholesterol, mmol/L	4.2 (3.8, 4.5)	3.9 (3.5, 4.5)	3.8 (3.5, 4.5)	4.2 (3.7, 4.4)	4.0 (3.6, 4.3)	3.7 (3.3, 4.2)
HDL cholesterol, mmol/L	1.1 (0.9, 1.2)	1.1 (1.0, 1.2)	1.1 (1.0, 1.3)	1.1 (0.9, 1.3)	1.1 (0.9, 1.3)	1.2 (1.1, 1.4)
LDL cholesterol, mmol/L	2.0 (1.7, 2.4)	2.3 (2.0, 2.8)	2.3 (2.0, 2.8)	2.0 (1.8, 2.4)	2.2 (1.9, 2.6)	2.2 (1.8, 2.5)
non-HDL cholesterol, mmol/L	3.1 (2.7, 3.5)	2.8 (2.4, 3.4)	2.7 (2.3, 3.2)	3.0 (2.6, 3.3)	2.9 (2.3, 3.1)	2.5 (2.1, 2.9)
Triglycerides, mmol/L	2.1 (1.4, 3.0)	1.0 (0.9, 1.2)	0.9 (0.6, 1.3)	2.1 (1.5, 2.7)	1.3 (0.9, 1.4)	0.8 (0.5, 1.0)

^1^ for diastolic, *n* = 89; ^2^ for percentiles, *n* = 44; ^3^ for percentiles, *n* = 128; ^4^ for percentiles, *n* = 130; ^5^ for LDL, *n* = 61; ^6^ for HDL, LDL, and triglycerides, *n* = 20. HDL, high density lipoprotein; LDL, low density lipoprotein.

**Table 4:** Cross sectional associations between breast milk total n-3 LC-PUFA content and blood pressure and blood lipid profile. Values presented are β (95% CI) for n-3 LC-PUFA (wt%) as exposure and outcome as indicated per row.

Group	Girls			Boys		
	4 Months	18 Months	36 Months	4 Months	18 Months	36 Months
**Blood pressure, *n***	76 ^1^	39 ^2^	108 ^3^	79	49	113 ^4^
Systolic BP, mmHg	2.4 (−10.3, 15.0)	−10.3 (−26.9, 6.3)	−0.5 (−7.5, 6.6)	−20.0 (−33.4, −6.7) ^B^	0.5 (−15.1, 16.1)	2.5 (−4.0, 9.0)
Diastolic BP, mmHg	3.6 (−8.2, 15.5)	−10.9 (−23.3, 1.4)	−0.2 (−5.3, 4.9)	−10.2 (−19.8, −0.5) ^A^	−0.8 (−16.5, 14.9)	4.7 (−0.3, 9.8)
Systolic BP, percentile	7.3 (−18.4, 33.0)	−21.2 (−41.6, −0.9) ^A^	1.2 (−16.5, 18.9)	−35.7 (−60.2, −11.3) ^B^	14.3 (−9.7, 38.3)	1.2 (−14.4, 16.7)
Diastolic BP, percentile	15.5 (−10.3, 41.2)	−4.8 (−17.2, 7.7)	0.6 (−8.6, 9.8)	−16.5 (−34.0, 1.0)	4.1 (−4.4, 12.6)	3.2 (−3.0, 9.4)
**Blood lipid profile, *n***	55 ^5^	18 ^6^	40	52	30	43
Total cholesterol, mmol/L	0.2 (−0.7, 1.0)	0.2 (−3.1, 3.6)	−0.8 (−2.2, 0.5)	0.2 (−0.8, 1.1)	0.7 (−1.0, 2.3)	−0.1 (−1.2, 1.1)
HDL cholesterol, mmol/L	−0.7 (−1.1, −0.3) ^B^	−0.2 (−1.1, 0.8)	−0.3 (−0.9, 0.3)	−0.1 (−0.6, 0.3)	−0.3 (−1.1, 0.5)	−0.2 (−0.7, 0.3)
LDL cholesterol, mmol/L	−0.1 (−1.0, 0.9)	0.7 (−3.2, 4.5)	−0.8 (−2.0, 0.3)	0.3 (−0.5, 1.1)	0.7 (−0.5, 2.0)	−0.2 (−1.1, 0.7)
Triglycerides, mmol/L	3.1 (1.0, 5.2)^B^	0.6 (−2.5, 3.8)	0.7 (−0.5, 1.9)	0.2 (−1.8, 2.1)	0.6 (−0.4, 1.6)	0.6 (−0.1, 1.3)

^1^ for diastolic, *n* = 75; ^2^ for percentiles, *n* = 38; ^3^ for percentiles, *n* = 103; ^4^ for percentiles, *n* = 110; ^5^ for LDL, *n* = 53, for triglycerides, *n* = 54; ^6^ for total cholesterol, *n* = 19. Analyses are adjusted for maternal pre-pregnancy BMI (in kg/m^2^), gestational age (in days), and educational level (in three categories; low, intermediate (reference), and high). ^A^ indicates *p* < 0.05 and ^B^ indicates *p* < 0.01.

## Supplementary Materials

The following are available online at http://www.mdpi.com/2072-6643/11/2/235/s1, Table S1: Associations between breast milk DHA content and blood pressure and blood lipid profile, Table S2: Associations between breast milk EPA content and blood pressure and blood lipid profile.
