# Supplementary material for: Content of n-3 LC-PUFA in Breast Milk Four Months Postpartum is Associated with Infancy Blood Pressure in Boys and Infancy Blood Lipid Profile in Girls
Source: Nutrients. 2019 Jan 22;11(2):235. doi: 10.3390/nu11020235 (PMC6412384; doi:10.3390/nu11020235)
Supplement: Supplementary file 1 [file nutrients-11-00235-s001.pdf]

nutrients

MDPI

Supplementary Table 1. Associations between breast milk DHA content and blood pressure and blood lipid profile.

	Girls			Boys		
	**4 months**	**18 months**	**36 months**	**4 months**	**18 months**	**36 months**
**Blood pressure, *n***	76 [1]	39 [2]	108 [3]	79	49	113 [4]
Systolic BP, *mmHg*	2.6 (-14.4, 19.6)	-12.0 (-34.2, 10.1)	-0.9 (-10.2, 8.4)	-24.7 (-41.1, -8.3)[B]	-1.8 (-22.4, 18.7)	4.3 (-4.0, 12.6)
Diastolic BP, *mmHg*	2.4 (-13.6, 18.5)	-14.9 (-31.3, 1.4)	-0.8 (-7.6, 6.0)	-13.8 (-25.6, -2.0)[A]	-4.4 (-24.9, 16.2)	6.5 (0.0, 12.9)[A]
Systolic BP, *percentile*	9.3 (-25.3, 44.0)	-27.1 (-54.2, -0.1)[A]	0.1 (-23.3, 23.5)	-43.2 (-73.3, -13.0)[B]	14.8 (-17.0, 46.7)	3.5 (-16.2, 23.3)
Diastolic BP, *percentile*	16.6 (-18.1, 51.3)	-6.8 (-23.3, 9.7)	-0.4 (-12.6, 11.8)	-22.0 (-43.5, -0.6)[A]	4.1 (-7.2, 15.3)	4.1 (-3.8, 12.0)
**Blood lipid profile, *n***	55 [5]	18 [6]	40	52	30	43
Total cholesterol, mmol/L	0.1 (-1.0, 1.3)	0.0 (-4.2, 4.3)	-1.0 (-2.9, 0.8)	0.3 (-0.9, 1.6)	0.9 (-1.3, 3.0)	-0.0 (-1.6, 1.5)
HDL cholesterol, mmol/L	-1.0 (-1.5, -0.4)[B]	-0.3 (-1.5, 0.9)	-0.5 (-1.3, 0.4)	-0.2 (-0.7, 0.4)	-0.4 (-1.5, 0.6)	-0.2 (-0.9, 0.5)
LDL cholesterol, mmol/L	0.0 (-1.2, 1.3)	0.3 (-4.6, 5.3)	-1.0 (-2.6, 0.7)	0.5 (-0.5, 1.5)	0.9 (-0.8, 2.5)	-0.2 (-1.5, 1.1)
Triglycerides, *mmol/L*	3.9 (1.1, 6.6)[B]	1.3 (-2.7, 5.3)	0.9 (-0.8, 2.5)	0.4 (-2.2, 3.0)	1.0 (-0.3, 2.3)	0.7 (-0.3, 1.7)

Analyses are adjusted for maternal pre-pregnancy BMI (in kg/m^2), gestational age (in days), and educational level (in three categories; low, intermediate (reference), and high).
Values presented are β (95% CI) for DHA (wt%) as exposure and outcome as indicated per row.
A indicates $p < 0.05$, and B indicates $p < 0.01$
[1] for diastolic, $n = 75$; [2] for percentiles, $n = 38$; [3] for percentiles, $n = 103$; [4] for percentiles, $n = 110$; [5] for LDL, $n = 53$; for triglycerides, $n = 54$; [6] for total cholesterol, $n = 19$

Supplementary Table 2. Cross sectional ssociations between breast milk EPA content and blood pressure and blood lipid profile.

	Girls			Boys		
	**4 months**	**18 months**	**36 months**	**4 months**	**18 months**	**36 months**
**Blood pressure, *n***	76 [1]	39 [2]	108 [3]	79	49	113 [4]
Systolic BP, *mmHg*	11.2 (-32.5, 54.8)	-46.6 (-106.6, 13.4)	0.5 (-25.5, 26.5)	-63.3 (-120.0, -6.7)[A]	18.0 (-34.8, 70.8)	-2.5 (-27.9, 22.9)
Diastolic BP, *mmHg*	27.5 (-13.2, 68.3)	-33.0 (-78.9, 12.9)	3.9 (-14.9, 22.8)	-18.1 (-59.0, 22.7)	19.8 (-33.2, 72.8)	10.9 (-8.9, 30.7)
Systolic BP, *percentile*	25.5 (-63.6, 114.6)	-78.8 (-154.1, -3.5)[A]	15.5 (-49.5, 80.5)	-123.3 (-226.3, -20.3)[A]	66.5 (-14.1, 147.0)	-15.1 (-75.1, 44.9)
Diastolic BP, *percentile*	75.4 (-12.8, 163.6)	-12.3 (-58.6, 34.1)	10.6 (-23.2, 44.4)	-33.2 (-107.1, 40.7)	20.0 (-8.6, 48.7)	10.0 (-14.1, 34.2)
**Blood lipid profile, *n***	55 [5]	18 [6]	40	52	30	43
Total cholesterol, mmol/L	1.0 (-1.7, 3.7)	2.7 (-8.5, 13.9)	-2.6 (-6.4, 1.2)	-0.2 (-3.5, 3.1)	2.0 (-3.7, 7.6)	-0.7 (-4.5, 3.2)
HDL cholesterol, mmol/L	-1.5 (-2.8, -0.1) [A]	0.4 (-2.9, 3.7)	-0.8 (-2.6, 0.9)	-0.5 (-1.9, 1.0)	-0.8 (-3.5, 2.0)	-0.8 (-2.5, 0.9)
LDL cholesterol, mmol/L	-1.0 (-3.9, 2.0)	5.5 (-7.3, 18.3)	-2.8 (-6.1, 0.5)	0.4 (-2.2, 3.1)	2.5 (-1.7, 6.8)	-1.0 (-4.1, 2.1)
Triglycerides, *mmol/L*	8.6 (1.6, 15.7) [A]	-1.8 (-12.7, 9.1)	2.2 (-1.2, 5.5)	-0.5 (-7.1, 6.2)	0.3 (-3.2, 3.8)	2.3 (-0.1, 4.8)

Analyses are adjusted for maternal pre-pregnancy BMI (in kg/m^2), gestational age (in days), and educational level (in three categories; low, intermediate (reference), and high).
Values presented are β (95% CI) for EPA (wt%) as exposure and outcome as indicated per row.
A indicates $p < 0.05$, and B indicates $p < 0.01$
[1] for diastolic, $n = 75$; [2] for percentiles, $n = 38$; [3] for percentiles, $n = 103$; [4] for percentiles, $n = 110$; [5] for LDL, $n = 53$; for triglycerides, $n = 54$; [6] for total cholesterol, $n = 19$
